# Effects of Ni and Cu Residuals on the Magnetic Properties and Microstructure of SmCo_5_ Magnets

**DOI:** 10.3390/ma15228226

**Published:** 2022-11-19

**Authors:** Muhammad Farhan Mehmood, Anas Eldosouky, Kristina Žužek Rožman, Sašo Šturm

**Affiliations:** 1 Jožef Stefan International Postgraduate School, Jamova 39, SI-1000 Ljubljana, Slovenia; 2 Department for Nanostructured Materials, Jožef Stefan Institute, Jamova 39, SI-1000 Ljubljana, Slovenia; 3 Magneti Ljubljana, d.d. Stegne 37, SI-1000 Ljubljana, Slovenia

**Keywords:** SmCo_5_ magnets, recycling, Ni/Cu coating, magnetic coercivity, microstructure, scanning electron microscopy (SEM)

## Abstract

The effect of Ni/Cu-coating residuals on the magnetic properties and microstructures of samarium–cobalt (SmCo_5_) magnets was studied. SmCo_5_ magnets with 0.0, 0.5, 1.0, 2.0, 3.0 and 4.0 wt.% of added Ni/Cu (85 wt.% Ni/15 wt.% Cu) were prepared using a conventional sintering route. The magnetic properties of the magnets were found to be consistent up to 2 wt.% Ni/Cu. Any further increase in the Ni/Cu content resulted in a significant reduction in the magnetic properties, to lower than values that would be commercially acceptable. SEM/EDS studies showed that two major phases, i.e., the SmCo_5_ matrix phase and Sm_2_O_3_ were present in all the sintered SmCo_5_ magnets. The presence of Sm_2_Co_7_ as a minor phase fraction was detected in the sintered SmCo_5_ magnets containing up to 2 wt.% Ni/Cu. A 2 wt.% Ni/Cu addition to magnets resulted in the presence of two new phases with compositions close to SmCo and Sm_2_Co_17_ in addition to SmCo_5_ and Sm_2_O_3_ as major phases in the SEM-observed microstructure. These newly formed phases are present in small fractions and are presumably homogenously distributed at the grain boundaries of the magnets. As they are known to act as nucleation sites for reverse magnetic domains, they effectively reduce the intrinsic grain boundary magnetic strength, leading to a drop in the coercivity. We concluded that the sintered SmCo_5_ magnets could be recycled with up to 2 wt.% Ni/Cu as a residual from the coating under our sintering and heat treatment conditions.

## 1. Introduction

Samarium (Sm) and cobalt (Co) are both economic and technically interesting. In addition to their use in permanent magnets, they are found in many other applications such as catalysts, batteries and lasers. Recently, the European Commission identified Sm, among other light-rare-earth elements (LREEs), as being second in terms of supply risk after the heavy rare earth elements. The European Commission will introduce Strategic Foresight Networks to develop robust evidence and scenario planning for raw materials supply, demand and use for strategic sectors in order to integrate the latest knowledge [1]. Recently, Co was also identified as one of the most important elements in terms of green and digital economic value [2,3]. The EU will need up to 5 times more cobalt in 2030, and almost 15 times more cobalt in 2050, to be used in permanent magnets for electric vehicle batteries, energy storage and digital technologies, compared with the current supply to the whole EU economy. If not addressed, this increase in demand will lead to supply issues [4]. Both direct and indirect recycling routes have been emphasized for compounds containing any element of supply risk and with economic importance. At the top are Sm-Co compounds. Indirect recycling consists of the individual separation of elements of interest after leaching. Direct recycling involves the reprocessing of compounds to be used in the same application as the original material.

Samarium–cobalt alloys are a fascinating class of magnetic materials that have an important role in permanent magnetism and modern technology. Since the discovery of high magnetocrystalline anisotropy in the SmCo_5_ compounds by Strnat and his colleagues in 1966 [5], SmCo_5_ magnets have been used in many applications, for example, space applications and electronics. Industrially, they usually have an intrinsic coercivity (H_Ci_) of 1592–2388 kA/m, a maximum energy product (BH_max_) of 130–245 kJ/m^3^ and typical remanence (B_r_) values of 0.8–0.96 T [6,7,8]. The microstructure of SmCo_5_ magnets consists of SmCo_5_ as the matrix phase with Sm-rich phases (Sm_2_Co_7_, Sm_5_Co_19_) and Sm-oxides as randomly distributed phases [9,10]. SmCo_5_ magnets are considered to be nucleation controlled, and the magnetic coercivity is related to the nucleation of reverse magnetic domains in regions where the anisotropy constant is reduced from the bulk value of the SmCo_5_ phase. This magnetization reversal takes place at the surfaces of the hard magnetic grains of SmCo_5_ or at grain boundaries and intergranular phases, which are also regions of reduced magnetocrystalline anisotropy. The lattice imperfections in the SmCo_5_ matrix might also be preferential sites to begin the magnetic reversal of the grain [11]. It was reported that the interplay between the domain wall pining and nucleation of reverse domains at the grain boundaries and grain boundary phases will affect the coercivity, which depends on a sintering temperature, heat treatment, chemical gradients and impurities [12,13,14,15,16]. A high sintering temperature has the effect of increasing the grain size, which reduces the coercivity, while the postheat treatment of sintered samples at 850–900 °C results in an enhanced coercivity due to the elimination of defects at the atomic level [12]. The presence of traces of the Sm_2_Co_17_ phase in the microstructures can serve as sites for the nucleation of reverse domains and therefore decrease the magnet’s coercivity [13].

SmCo_5_ sintered magnets are prepared industrially by milling the as-cast alloy to produce a powder of ≤20 µm particle size. This powder is then aligned, pressed and sintered [17,18]. Sintered magnets are usually coated with a mixture of Ni and Cu against corrosion and to improve the mechanical strength during the magnet’s handling and final operation. For direct recycling, the magnets should be converted to the powder form; for example, by recasting and milling or hydrogen decrepitation and milling. Although the SmCo_5_Ni_5-x_ and SmCo_5_Cu_5-x_ systems can have very high H_Ci_ values of up to 20 T measured at room temperatures [19,20], several studies showed that the Ni/Cu-coating residuals have a detrimental effect on the coercivity of a recycled magnet prepared by either of the above processing routes [21].

The goal of this study was to determine the maximum concentration of Ni/Cu-coating residuals present in the recycled SmCo_5_ powder so that the fabricated, recycled magnets would still have acceptable magnetic properties. Six different mixtures of SmCo_5_ powders with different Ni/Cu concentrations were prepared and conventionally sintered to produce the magnets. The differences in density, oxygen content, magnetic properties, microstructure and crystal structure of the sintered magnets with different Ni/Cu contents are discussed. The normal coercivity (Hc) is the magnetic field required to reduce the magnetic flux to zero, while the intrinsic coercivity (Hci) is the magnetic field required to reduce the magnetization to zero. It can be measured either empirically or by mathematical analysis. The coercivity of a magnetic material is expressed by the magnetization curve. This can also be called a magnetic hysteresis loop. The hysteresis loop shows how the external magnetizing force and the induced magnetic flux density are related.

For the validity of the comparison, the sintering temperature and heat-treatment profiles were the same for all samples.

## 2. Materials and Methods

The as-cast Sm-Co alloy with a chemical composition of 36.23 wt. % Sm (18.20 at.% Sm) and 63.77 wt.% Co (81.81 at.% Co) was crushed and milled in a nitrogen atmosphere to produce powder with a particle size of 4–20 µm. Cu and Ni powders (>99.5% purity, Sigma-Aldrich, USA) with a particle size of less than 5 µm were used. First, a mixture of 85 wt.% Ni and 15 wt.% Cu was prepared. This chemical composition of Ni and Cu was chosen to simulate the coating from the sintered SmCo_5_ magnet. However, the exact ratio might vary depending on the application procedure and the magnet volume. In the present study, six different mixtures of milled SmCo_5_ powder with 0, 0.5, 1.0, 2.0, 3.0 and 4.0 wt.% Ni/Cu were prepared. All the mixtures of SmCo_5_ and Ni/Cu were prepared inside a glove box to prevent the oxidation of the SmCo_5_ magnet powders. After preparation, the powders were mixed in a tubular mixer for 4 h to ensure complete homogenization. All the prepared mixed SmCo_5_ + Ni/Cu samples were sintered at 1180 °C for 3 h, cooled down to 880 °C at 1 °C min^−1^ and held for 2 h at this temperature for heat treatment under a partial pressure of 100 mbar of argon (Ar) gas and then cooled to room temperature. The density (ρ) of the samples were measured by Archimedes’ principle.

The oxygen (O) content in the samples was measured with an oxygen/nitrogen analyzer (Eltra ON 900, Retsch-Allee, Haan, Germany). For the analysis, 0.2–0.3g of sample were taken in a graphite crucible. The sample was melted in an inert atmosphere of helium (He) gas, carbon from the graphite crucible chemically reacted with the oxygen present in the sample to produce carbon monoxide (CO) gas, which was measured to calculate the oxygen content. The Magnet-Physik’s Permagraph (Köln, Germany) with a maximum applied field of 1500 kA/m was used to measure the demagnetization behavior of the previously magnetized samples. An opposite magnetic field was applied to the previously magnetized sample and the polarization of the sample was measured to draw the second quadrant of the hysteresis loop. The instrument required a symmetrical sample with two parallel faces. For the microstructure characterization, a small amount of the magnet powder was mounted in an epoxy mixed with a suitable hardener. The mounted sample was then grinded using a series of silicon carbide grinding sheets having grit sizes of P500, P800, P1000, P2000 and P4000. Then, the sample was polished using cloth sheets and a polycrystalline diamond paste (3 µm and ¼ µm). A Struers Tegrapol 15 automatic metallurgical polisher was used for the preparation of the microscope samples for the material’s evaluation. During the preparation steps, the samples were continuously checked with a Nikon Eclipse E600POL optical microscope to ensure the grinding efficiency and the removal of scratches. To avoid the charging of the sample by the electron beam, a less-than-10-nm carbon coating was applied using a BAL-TEC SCD 005 Cool Sputter Coater (Switzerland). The carbon coating was applied by evaporation using a heated carbon thread under vacuum. The specimens were then examined for microstructures using a Helios NanoLab™ 650 scanning electron microscope equipped with an energy-dispersive spectrometer (EDS). The elemental distribution was identified with an EPMA-1720 electron probe microanalyzer (EPMA). An operating voltage of 20 kV was used for the microscope.

## 3. Results and Discussion

### 3.1. Measurements of Density and Oxygen Content

The density measurement data and oxygen content for all the sintered SmCo_5_ magnet samples are reported in Table 1. The density values of the sintered SmCo_5_ with 0–3.0 wt.% Ni/Cu were found to be approximately 8.30 g cm^−3^, but a value of 8.20 g cm^−3^ was observed for the sample containing 4.0 wt.% Ni/Cu. The theoretical density of a pure SmCo_5_ magnet is 8.5 gcm^−3^ [20]. The oxygen (O) content in all samples was found to be 0.31–0.325 wt.% O (Table 1). The oxygen content in all the samples was less than 0.35 wt.%, which is acceptable for a SmCo_5_ magnet [11].

### 3.2. Magnetic Properties

**Figure 1:**
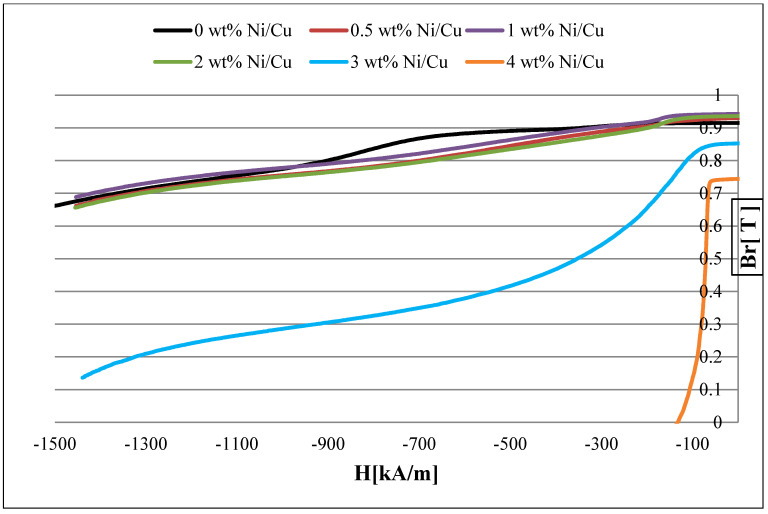
Demagnetization curves for the SmCo_5_ magnets with 0–4 wt% Ni/Cu contents.

The demagnetization curves are shown in Figure 1. Because of the magnetometer applied field limitations, we based our mechanism explanations on the demagnetization curve trends, which showed obvious differences in coercivities after simple interpolation. It is true that the full hysteresis loops would provide an even better insight into the demagnetization behavior, but for that, larger demagnetization fields provided via a different magnetometer apparatus would be necessary. Table 2 summarizes the remanence (B_r_), energy product (BH_max_), normal magnetic coercivity (Hc) and intrinsic coercivity (Hc_i_) for the sintered SmCo_5_ magnets. H_c_ or H_ci_ is called normal coercivity. It is the point on the BH curve where the magnet’s internal field is completely cancelled out by the opposing field. In other words, the net flux density is now zero because the opposing field is equal and opposite of the magnet’s own field. At this point, the demagnetization is still recoverable because the magnet has not lost its polarity. Up to 2 wt. % Ni/Cu, the magnets showed good magnetic properties with similar demagnetization behaviors. The values of BH_max_ were higher than 150 kJ/m^3^ and the H_C_ values were higher than 640 kA/m. A small increase in the B_r_ value from 0.92 T to 0.94 T was observed with an increase in the Ni/Cu content up to 2 wt. %, which might be due to a decrease in the Sm_2_Co_7_ phase content with the Ni/Cu increase, as will be discussed in the next section.

The presence of a large amount of the Sm_2_Co_7_ phase in the 0 wt. % Ni/Cu might also be the reason for the small shoulder in its demagnetization curve. For the 3 wt. % Ni/Cu magnet, there was a significant decrease in B_r_ from 0.92–0.94 T to 0.85 T. Moreover, the demagnetization curve had a noticeable shoulder with the decrease in the H_C_ value to 382 kA/m (Figure 1). This indicates the formation of new phases in the microstructure with a negative effect on the magnetic properties. Consequently, this magnet is unsuitable for applications. Increasing the Ni/Cu concentration further results in the loss of almost all of the magnet’s coercivity to a value less than 150 kA/m, with a very low BH_max_ value of 23.98 kJ/m^3^ (Table 2).

### 3.3. Microstructure Characterization

**Figure 2:**
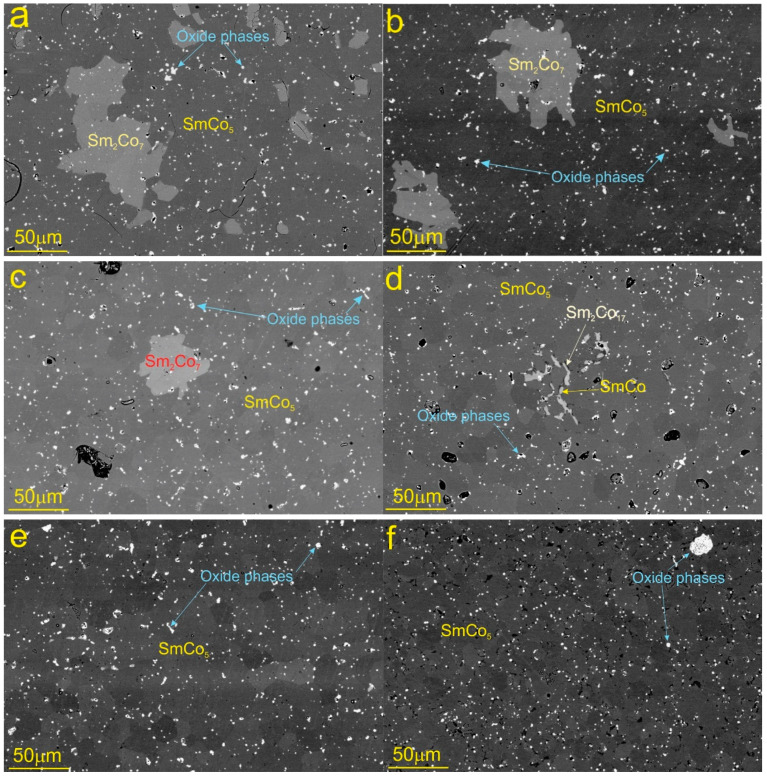
BSE images for the samples with (**a**) 0 wt%, (**b**) 0.5 wt%, (**c**) 1 wt%, (**d**) 2 wt%, (**e**) 3 wt% and (**f**) 4 wt% Ni/Cu, from left to right. For the magnets with Ni/Cu ≥ 3 wt%, the SmCo and Sm_2_Co_17_ phases were homogeneously distributed in the microstructure, mainly at the grain boundaries, in contrast to the magnet with Ni/Cu of 2 wt% and more where the phases appear only as random clusters.

Systematic SEM/EDX analyses of the magnets were performed to correlate the coercivity decrease and the resulting microstructures with different Ni/Cu contents. Figure 2 shows BSE images of the magnets with 0 wt. % to 4 wt.% Ni/Cu. Representative EDX analyses with the corresponding nominal compositions for the phases in the SmCo_5_ magnets with 0–2 wt.% Ni/Cu and 4 wt.% Ni/Cu contents are shown in Table 3. For the sintered SmCo_5_ magnets with 2 wt% Ni/Cu, the microstructure is similar (Figure 2a,b) as reported in the literature [9]. The magnets consist of the SmCo_5_ matrix phase with a random distribution of Sm-rich phases, Sm_2_Co_7_, Sm oxides and pores that might explain the lower density measured for this sample. The volume ratio of the Sm_2_Co_7_ phase in the microstructures decreased with increasing Ni/Cu content. The Sm_2_Co_7_ phase disappeared for the samples with 2, 3 and 4 wt.% Ni/Cu, as observed from the microstructure study (Figure 2d,f). However, this magnet was still showing good magnetic properties, similar to the samples with lower Ni/Cu concentrations. These phases were consistently measured in the sample with 3 wt.% and 4 wt.% of Ni/Cu to be a Sm-rich phase near to the SmCo phase (light gray) and the Co-rich phase with a composition near to Sm_2_Co_17_ (dark gray), with partial solubility of the Ni and Cu. The volume fraction of these new phases increased with an increase in the Ni/Cu content. For the 3 wt. % and 4 wt. % Ni/Cu samples, the SmCo and Sm_2_Co_17_ phases formed at the grain boundaries and as intergrown clusters. The Sm_2_Co_7_ phase was not formed in the magnet with 2% Ni/Cu; however, this magnet had good magnetic properties, similar to the samples with a lower Ni/Cu concentration. In some parts of the microstructure of the 2 wt.% Ni/Cu magnet, a new phase of very small fractions (dark grey phase) started to form at the grain boundaries. The distribution of this new phase was shown to increase with an increase in the Ni/Cu. Using the EDX, the composition of the phase was measured in the sample with 4 wt.% Ni/Cu to be a Co-rich phase with a composition close to Sm_2_Co_17_. The fraction of the phase was much higher for the magnet with 4 wt.% Ni/Cu, and this is believed to be the reason for the magnet losing almost all its coercivity. It was concluded that the presence of the impurity phase at the grain boundaries and therefore the decrease in the pinning strength is the main reason for the coercivity drop in the magnets with 4wt.% Ni/Cu in comparison to pristine magnets having the composition of SmCo_5_, Sm_2_CO_7_ and the Sm oxide.

It is hypothesized that the newly formed phases contribute to a decrease in the pinning strength of the reverse domains in the microstructure, which is translated to the overall coercivity drop. Although some amounts of the SmCo and Sm_2_Co_17_ phase can be observed in the magnets with 2 wt.% Ni/Cu fractions, mainly as intergrown clusters, they do not significantly affect the demagnetization behavior of the magnet. This is in striking contrast to the magnets with the highest Ni/Cu contents (4 wt. % Ni/Cu), where these phases could not be observed in the microstructure given the spatial resolution. From these results it is concluded that Ni and Cu do not form any discrete phases with either Sm or Co, as they only replace some of the Co atoms, mainly in the crystal structure of the Sm-Co phases. Xu et al. [22] reported a new design of using the Sm_2_Co_7_ nanophase to induce low-temperature hot deformation to prepare SmCo_5_ magnets. The Sm_2_Co_7_ nanophase can promote the grain rotation and grain boundary sliding of the SmCo_5_ phase, thereby coordinating the deformation of the two phases and avoiding local stress concentrations, and finally obtaining a high coercivity magnet with a good c-axis texture under low-temperature hot deformation. Bartlett et al. [23] found that the performance of the SmCo_5_ magnet deteriorated mainly due to the easier nucleation of the reverse domain due to the precipitation of Sm_2_Co_17_. Chen et al. [18] observed that the 1:5 phase and 2:7 phase coexisted in the spark-plasma-sintered SmCo_5_ magnet, and a 2:17 phase appeared when the temperature rose to 1050 °C. The best magnetic properties were obtained for the magnets prepared at 1000 °C, with remanence (Br) = 0.45 T, intrinsic coercivity (Hci) = 985 kA/m, and maximum energy density (BH)max = 37 kJ/m^3^. The microstructure analysis showed that the high density of the magnet and the uniform distribution of the hard magnetic phase are the main reasons for the excellent magnetic properties. The anisotropy of Sm_2_Co_17_ is lower than that of SmCo_5_ by a factor of about four, but the cellular microstructure of the 2:17 magnets is ideal for coercivity development [24,25,26,27,28].

Based on the EDX analysis of the elements, the nominal composition of new phases such as SmCo_4.75_Ni_0.25_Cu_0.04_, Sm_2_Co_16.5_Ni_0.5_Cu_0.08_ and SmCo_1-x_Ni_x_ can be formulated in the magnet samples containing >2 wt.% Ni/Cu contents by saying the new phases are formed on the account of the Sm_2_Co_7_ phase. Increasing the Ni/Cu concentration resulted in the change in the SmCo_5_ and Sm_2_Co_7_ phase crystal lattice with the partial substitution of Co by Cu and Ni to form new phases. This is known from the literature, as the substitution of Co with Cu in the sintered SmCo_5_ magnet has already been reported [29,30,31], and this occurs because of the similar atomic radii of Co (1.52 Å), Ni (1.49 Å) and Cu (1.45 Å) and the same ionic radii of 1.35 Å for Co, Ni and Cu [32,33]. With the increase in the Ni/Cu concentration, the oxygen content increases and forms an Sm-oxide phase, which also results in a decrease in the overall magnetic properties.

## 4. Conclusions

For sintered SmCo_5_ magnets to be recycled with no significant reduction in the magnetic properties, the concentration of Ni/Cu-coating residuals should not be more than 2 wt.% for our sintering and heat-treatment conditions. The recycled magnet with a higher Ni/Cu content (4 wt.% Ni/Cu) had a drastic reduction in the magnetic properties, which might be due to newly formed phases, i.e., SmCo and Sm_2_Co_17_, dispersed in the microstructure, contributing to a decrease in the pinning strength of the reverse domains in the microstructure, which is translated to the overall coercivity drop. As they are known to act as nucleation sites for the reverse domains, they effectively decrease the intrinsic grain-boundary magnetic strength, controlling the overall coercivity. We conclude that the recycling of permanent magnets for the sustainable production of permanent magnets with a sintering route is possible.

## Figures and Tables

**Table 1:** Density and oxygen contents of sintered samples of SmCo_5_ with 0–4 wt% Ni/Cu.

Sintering Conditions	Density (g/cm^3^)	Oxygen (wt%)
100 wt% SmCo_5_	8.30	0.310
99.5 wt% SmCo_5_ + 0.5 wt% Ni/Cu	8.30	0.325
99.0 wt% SmCo_5_ + 1.0 wt% Ni/Cu	8.30	0.334
98.0 wt% SmCo_5_ + 2.0 wt% Ni/Cu	8.30	0.325
97.0 wt% SmCo_5_ + 3.0 wt% Ni/Cu	8.30	0.314
96.0 wt% SmCo_5_ + 4.0 wt% Ni/Cu	8.20	0.315

**Table 2:** Magnetic properties for the magnets with 0–4 wt% Ni/Cu.

Sintering Conditions	BH_max_ [kJ/m^3^]	B_r_ [T]	H_Ci_ [kA/m]	H_C_ [kA/m]
100 wt% SmCo_5_	175.1	0.92	>1500	691.8
99.5 wt% SmCo_5_ + 0.5 wt% Ni/Cu	154.4	0.93	>1500	646.0
99.0 wt% SmCo_5_ + 1.0 wt% Ni/Cu	159.3	0.94	>1500	660.1
98.0 wt% SmCo_5_ + 2.0 wt% Ni/Cu	150.3	0.94	>1500	641.7
97.0 wt% SmCo_5_ + 3.0 wt% Ni/Cu	130.5	0.85	>1500	381.8
96.0 wt% SmCo_5_ + 4.0 wt% Ni/Cu	23.98	0.74	132.8	101.3

**Table 3:** EDX analysis for the phases in the sintered SmCo_5_ magnet samples with and without Ni/Cu.

Phase	EDX Analysis (at %)					Sm:Co Ratio	Nominal Phase Composition
	Sm	Co	O	Ni	Cu		
***Sintered SmCo_5_ magnet with 0 wt.% Ni/Cu***							
Matrix phase	16.8	81.7	1.5	-	-	1:5	SmCo_5_
Grey phase	22.4	75.9	1.8	-	-	2:7	Sm_2_Co_7_
White small spots	35.7	11.0	53.3	-	-	*	Sm_2_O_3_
***Sintered SmCo_5_ magnet with 2 wt.% Ni/Cu***							
Matrix Phase	16.2	80.3	0.9	2.3	0.3	1:5	SmCo_5_
Light Gray Phase	49.3	48.1	1.5	1.3	0.1	1:1	SmCo
Dark Gray Phase	11.2	85.2	0.8	2.4	0.4	2:17	Sm_2_Co_17_
White small spots	30.7	17.0	52.0	0.3	N.D.	*	Sm_2_O_3_
***Sintered SmCo_5_ magnet with 4 wt.% Ni/Cu***							
Matrix Phase	16.0	78.0	1.1	4.2	0.6	1:5	SmCo_5_
White small spots	36.0	5.0	58.5	0.4	0.1	*	Sm_2_O_3_

N.D. = Not detectable. * = Sm:O ratio is 2:3 indicating the formation of Sm_2_O_3_ as small white spots in the microstructure.
